# Essure Microinsert Abdominal Migration after Hysteroscopic Tubal Sterilization of an Appropriately Placed Essure Device: Dual Case Reports and Review of the Literature

**DOI:** 10.1155/2015/402197

**Published:** 2015-11-18

**Authors:** Shadi Rezai, Meghan LaBine, Hunter Azdel Gomez Roberts, Isamarie Lora Alcantara, Cassandra E. Henderson, Malvina Elmadjian, Dilfuza Nuritdinova

**Affiliations:** ^1^Department of Obstetrics and Gynecology, Lincoln Medical and Mental Health Center, Bronx, NY 10451, USA; ^2^School of Medicine, St. George's University, St. George's, Grenada

## Abstract

*Background*. The Essure device is a method of permanent sterilization widely used in the US that has proven to be safe and effective in most cases. However, there have been reports of device migration that have led to failed tubal occlusion as well as several other serious complications resulting from the presence of the device in the abdominal cavity.* Case*. This paper represents two cases of failed tubal occlusion by an appropriately placed Essure device without signs or symptoms of further complications related to device migration.* Conclusion*. Although there have only been 13 reported cases of abdominal device migration since November 2014, this case indicates that the actual number may be higher than reported since it is possible for migration to occur without additional complications. In the majority of reported cases of abdominal migration a major complication requiring surgical correction occurred, such as adhesions, small bowel obstruction, bowel perforation, or persistent pelvic pain. To avoid these complications it is recommended that migrating implants be removed; however, this case also represents an example of when a migrating device may remain* in situ* in an asymptomatic patient.

## 1. Background

The Essure pbc (Permanent Birth Control System) device is a dynamic expanding microinsert that stimulates benign tissue growth when placed in the proximal section of the fallopian tube, eventually occluding the tube. The device was approved in 2002 as a means of permanent sterilization by the US Food and Drug Administration (FDA), and initial reports showed a high rate of safety and patient acceptability [1–4].

The benefits of the Essure hysteroscopic tubal occlusion/sterilization procedure are unique from other permanent birth control methods because it is hormone-free and does not require a skin incision [4, 5]. Essure placement can be done as an office based procedure since patients do not require general anesthesia, allowing recovery to be quicker than other types of sterilization [4, 5]. Most women can go home 45 minutes after the procedure and return to normal activity within one to two days [4, 5].

Upon placement of Essure, ideally 3 to 8 outer coils of the expanded Essure microinsert should be trailing into the uterus (Figure 6). A follow-up hysterosalpingogram (HSG) three months after placement of the device is a safe method of confirming satisfactory placement and tubal occlusion [4, 6, 7]. Unless the microinsert has a trailing length that is greater than 18 expanded outer coils, the microinsert should be left in place [2]. Two-dimensional ultrasound (2DUS) can also be used and is more time-efficient and equivalent to three-dimensional ultrasound (3DUS) in locating Essure contraceptive microinserts to ensure correct placement at the time of initial insertion and for periodic checks later [8, 9].

The Essure procedure is 99.83 percent effective at preventing pregnancy when used according to the approved instructions for use based on five-year clinical study data [4, 5]. Despite the benefits of Essure hysteroscopic tubal sterilization, there are potential risks of this procedure as well. It is important to remember that no form of birth control should be considered 100 percent effective. Not all women will achieve successful placement of both inserts. Studies have shown that up to 9.6 percent of women could become pregnant within 10 years of undergoing hysteroscopic sterilization [10], and abdominal migration and tubal perforation are rare but recognized complications [3, 10]. Of the approximately 50,000 Essure insertion procedures performed between 1997 and 2005, there were 64 reports of unintended pregnancies to the manufacturer, most of which were attributed to failure to use alternative birth control methods prior to confirmation that the device had expanded to fully occlude the fallopian tubes [11]. This report, however, documents a case of failure of tubal occlusion of a previously appropriately placed Essure device [1] due to Essure microinsert abdominal migration that did not result in major complications requiring surgical intervention [3]. The patient was asymptomatic prior to pregnancy, indicating that there may be more unreported cases of device migration. These risks and complications should be taken into account when considering Essure as a method of sterilization.

## 2. Presentation of Case Number 1

The patient is a 29-year-old Hispanic, para 3003, with no past medical or surgical history, who was satisfied with her parity and electively underwent an uncomplicated hysteroscopic tubal sterilization with Essure device in January 2013.

**Figure 1:**
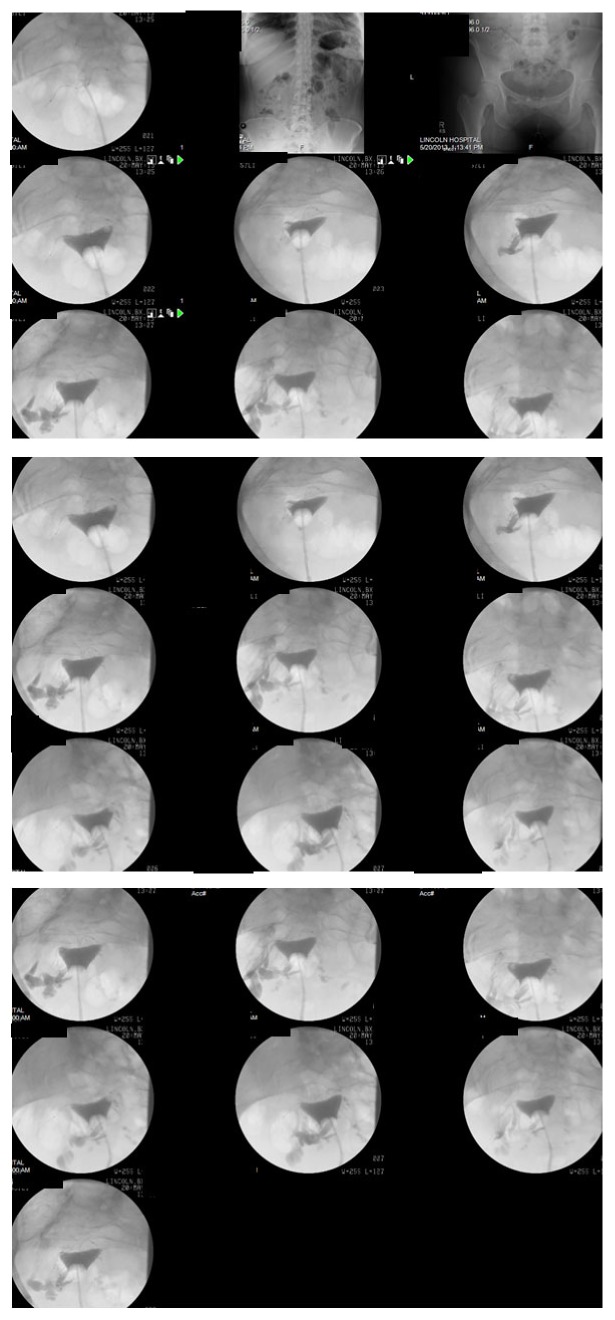
Patient Number 1, hysterosalpingogram DX (HSG) on 5/23/13, showing proper Essure placement with bilateral tubal occlusion with no spillage of the contrast.

Intraoperatively, eleven coils were seen outside the right ostium and four coils were seen at the left ostium upon Essure placement. Her 6-month follow-up HSG, in May 2013, showed proper Essure placement with bilateral tubal occlusion with no spillage of the contrast materials (Figure 1).

However, in January 2014, the patient was evaluated for low abdominal pain. Ultrasound at that time showed an intrauterine pregnancy (IUP) with 2.42 cm Crown Rump Length (CRL), corresponding to 9 weeks and 2 days of gestational age (GA).

**Figure 2:**
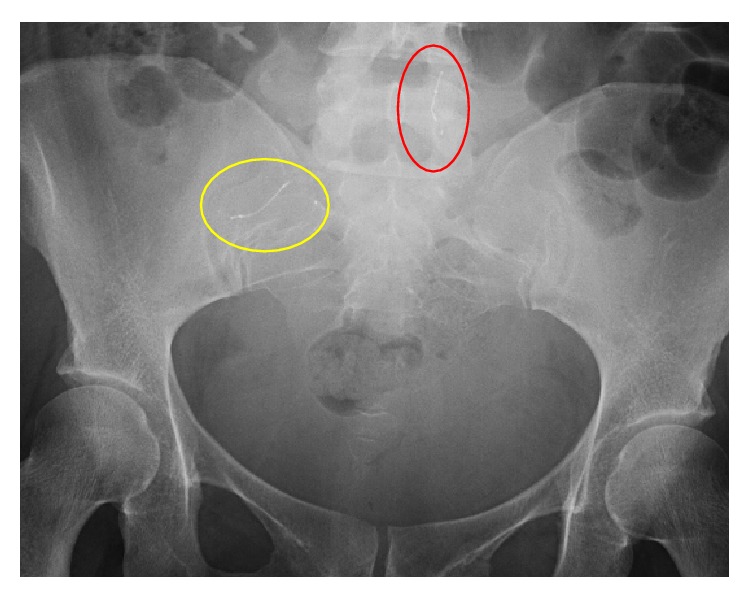
Patient Number 1, postpartum pelvic X-ray 8/30/2014: Essure (circled in red) in the abdomen.

**Figure 3:**
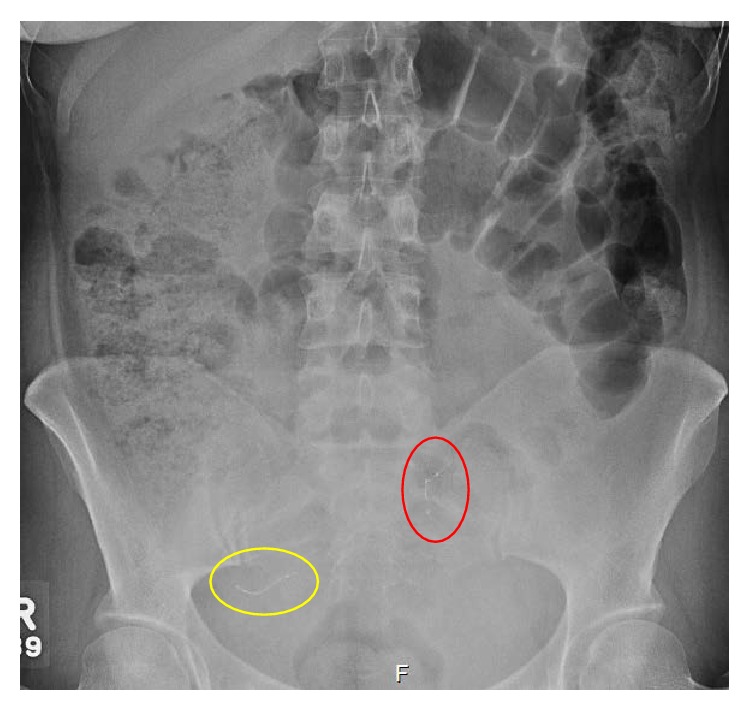
Patient Number 1, postpartum abdominal X-ray 8/30/2014: Essure (circled in red) in the abdomen.

Patient had an uncomplicated spontaneous vaginal delivery in November 2014 and underwent postpartum bilateral salpingectomy. It was noted intraoperatively that no Essure microinserts were identified in the fallopian tubes. Postpartum X-ray of abdomen and pelvis identified Essure coils in the peritoneal cavity (Figures 2 and 3). Patient declined laparoscopy for removal of Essure microinsert.

## 3. Presentation of Case Number 2

The patient is a 35-year-old para 6016, Hispanic female, with history of appropriately placed Essure device in 2011. Intraoperatively, six coils were seen outside the right ostium and one coil was seen at the left ostium upon uncomplicated Essure procedure placement. Patient did not come back for 3-month post-Essure hysterosalpingogram.

**Figure 4:**
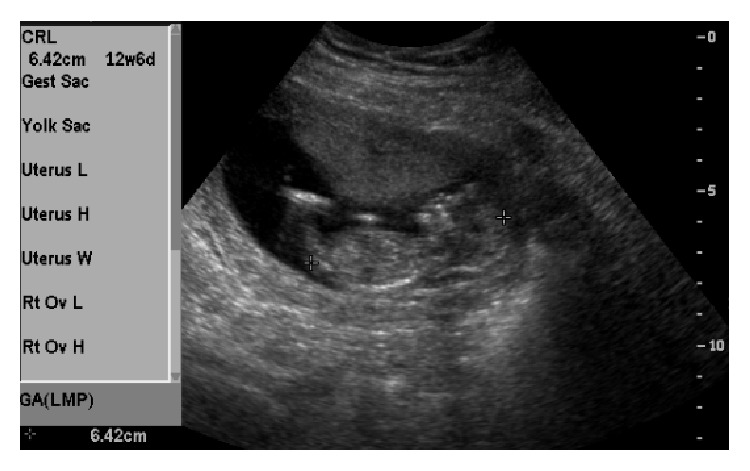
Patient number 2, pelvic sonogram on 4/2/15: showing IUP at 12 6/7 wks, while both Essure inserts are still in place.

**Figure 5:**
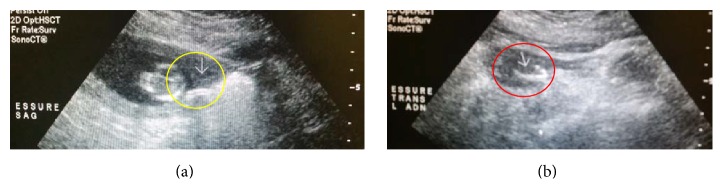
Patient number 2, pelvic sonogram on 4/2/15: showing right Essure inserts, sagittal view (yellow circle), and left Essure insert, transverse view (red circle).

The patient became pregnant in 2015 while both Essure inserts were still present in both fallopian tubes (Figures 4 and 5) and had an uncomplicated pregnancy and full term vaginal delivery. The patient underwent postpartum bilateral distal salpingectomy, fimbriectomy, and umbilical hernia repair, after which the Essure device was not seen in either of the tubes.

**Figure 6:**
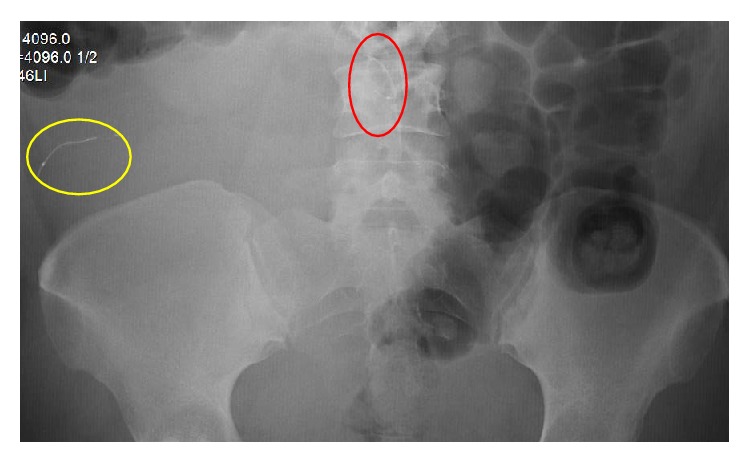
Patient Number 2, postpartum pelvic X-ray 10/13/15: Essure (circled in yellow) in the right upper quadrant around the liver. The 2nd Essure insert (circled in red) is seen by lumbar vertebra.

X-ray of the abdomen and pelvis only showed one Essure insert in the right upper quadrant near the liver, while the other Essure insert was at the level of lumbar vertebra (Figure 6). The patient opted for Essure removal procedure with diagnostic laparoscopy and removal of Essure device.

## 4. Discussions

Abdominal displacement of the Essure pbc is a very rare complication. Only 14 cases had been reported out of the 750,000 devices that had been placed to date, according to the US Food and Drug Administration. Case reviews suggest that several factors influence the likelihood of complications, including physician experience in placement and anatomical anomalies in the patient [3].

Device displacement has been reported in three cases complicated by laterally sited ostia, tubal resistance, or endometrial adhesions but displacement was also reported in five women who had uneventful procedures, such as our patient. In the majority of cases of abdominal displacement, the patients were asymptomatic and migration of the device was diagnosed at the 3-month follow-up HSG [3, 4, 7, 12–14]. Despite being asymptomatic, most physicians, and our second patient in this case report, elected to remove the displaced device. Generally this is easily done laparoscopically, with the exception of the cases reported by Mantel et al. and Belotte et al. where the device was implicated in causing small bowel obstruction and perforation [3, 12, 13]. Removal has therefore become the standard practice in management of device migration.

The first patient in this case, however, declined laparoscopic removal of the Essure device. This was observed to be a safe option by Kerin et al. [3, 15] who reported three cases of migrating Essure devices that were left* in situ* after noting that the pelvic organs were healthy and normal. They did not report any later complications for those patients [2, 3].

The cause of device migration is not fully understood in all cases. Some cases have been reported where displacement was due to uterine or tubal perforation, but there have also been cases where no signs of perforation were seen, as in the first patient highlighted in our report. It is suspected that migration in those cases occurred through the natural opening in the fallopian tube [3, 7, 12, 13].

In other reported cases it was observed that the left implant is more commonly implicated in migration than the right, suggesting that proper insertion of the device into the left tube by right-handed physicians may be difficult [3]; however, this is unproven and not likely to be the cause in the subject of this report because both implants failed to occlude the fallopian tubes.

## 5. Conclusion

As of November 2014, there were only 14 cases of Essure abdominal migration in the literature [3]. In some cases it has caused a severe adverse event such as adhesion [13], pelvic pain [14], small bowel obstruction [15], or bowel perforation [12] requiring major surgery [3, 12]. There have been cases of tubal perforation after Essure placement for which ultrasound failed to diagnose [10]. However, in most cases, abdominal displacement of the microinsert is asymptomatic and does not induce tissue damage, such as in the patients indicated in this report [3]. This reinforces the importance of follow-up HSG six months after the Essure device has been placed, in addition to the three-month follow-up that is currently standard practice, in order to gain better insight into the incidence of device migration.

Incorrect position of Essure microinserts can be seen postoperatively, when the initial placement procedure was difficult. In these cases, it is recommended to perform a transvaginal ultrasound (TVU) or pelvic X-ray 4 weeks after the procedure or after the first vaginal bleeding. It is also recommended that removal of the migrating device should be performed as soon as possible [3]. Moreover, during presterilization counseling, the patient should also be completely informed about the risks of these rare but relevant complications, as well as about the surgical interventions that could be required to solve the complication [3]. Even in the setting of appropriately placed Essure microinserts patient may have persistent postprocedure pain, which is an indication that the microinserts should be removed surgically [14]. However, if the patient is asymptomatic, pelvic organs appear healthy, and the patient declines device removal; evidence has shown that the patient may continue to live comfortably and without complications from the displaced device [2, 3].
